# Advancing PHBV Biomedical Potential with the Incorporation of Bacterial Biopigment Prodigiosin

**DOI:** 10.3390/ijms24031906

**Published:** 2023-01-18

**Authors:** Marijana Ponjavic, Ivana Malagurski, Jelena Lazic, Sanja Jeremic, Vladimir Pavlovic, Nevena Prlainovic, Vesna Maksimovic, Vladan Cosovic, Leonard Ionut Atanase, Filomena Freitas, Mariana Matos, Jasmina Nikodinovic-Runic

**Affiliations:** 1 Institute of Molecular Genetics and Genetic Engineering, University of Belgrade, Vojvode Stepe 444a, 11042 Belgrade, Serbia; 2 Faculty of Agriculture, University of Belgrade, Nemanjina 6, 11080 Belgrade, Serbia; 3 Faculty of Technology and Metallurgy, University of Belgrade, Karnegijeva 4, 11000 Belgrade, Serbia; 4 Vinca Institute of Nuclear Sciences, University of Belgrade, National Institute of the Republic of Serbia, Mike Petrovića Alasa 12-14, 11000 Belgrade, Serbia; 5 Institute of Chemistry, Technology and Metallurgy, University of Belgrade, Njegoseva 12, 11000 Belgrade, Serbia; 6 Faculty of Dental Medicine, “Apollonia” University of Iasi, 700511 Iasi, Romania; 7 Academy of Romanian Scientists, 050045 Bucharest, Romania; 8 i4HB—Institute for Health and Bioeconomy, School of Science and Technology, NOVA University Lisbon, 2819-516 Caparica, Portugal; 9 UCIBIO—Applied Molecular Biosciences Unit, Department of Chemistry, School of Science and Technology, NOVA University Lisbon, 2819-516 Caparica, Portugal

**Keywords:** prodigiosin, polyhydroxyalkanoate, PHBV, anticancer activity, film, drug delivery, biopolymer crystallinity, biocompatibility

## Abstract

The quest for sustainable biomaterials with excellent biocompatibility and tailorable properties has put polyhydroxyalkanoates (PHAs) into the research spotlight. However, high production costs and the lack of bioactivity limit their market penetration. To address this, poly(3-hydroxybutyrate-co-3-hydroxyvalerate) (PHBV) was combined with a bacterial pigment with strong anticancer activity, prodigiosin (PG), to obtain functionally enhanced PHBV-based biomaterials. The samples were produced in the form of films 115.6–118.8 µm in thickness using the solvent casting method. The effects of PG incorporation on the physical properties (morphology, biopolymer crystallinity and thermal stability) and functionality of the obtained biomaterials were investigated. PG has acted as a nucleating agent, in turn affecting the degree of crystallinity, thermal stability and morphology of the films. All samples with PG had a more organized internal structure and higher melting and degradation temperatures. The calculated degree of crystallinity of the PHBV copolymer was 53%, while the PG1, PG3 and PG3 films had values of 64.0%, 63.9% and 69.2%, respectively. Cytotoxicity studies have shown the excellent anticancer activity of films against HCT116 (colon cancer) cells, thus advancing PHBV biomedical application potential.

## 1. Introduction

PHAs are bacterial polyesters produced under unbalanced growth conditions (i.e., excess carbon and limitation of inorganic nutrients) as a depo of carbon and energy [1]. Depending on the number of C-atoms in the monomeric unit, they are classified into short-chain-length (scl-PHAs) (3 to 5 C-atoms) and medium-chain-length (mcl-PHAs) (6 to 14 C-atoms) polyhydroxyalkanoates, with scl-PHAs being highly crystalline and brittle, while mcl-PHAs are semi-crystalline, elastomers. The monomers are combined into homo-(e.g., poly(3-hydroxybutirate), PHB) or co-polymers (e.g., poly(3-hydroxybutyrate-co-3-hydroxyvalerate), PHBV) with different chain lengths, which gives this class of biomolecules structural diversity and, subsequently, a wide range of physicochemical properties [2]. In addition, their properties can be tuned to specific requirements through variations in monomer composition, polymer chain length and branching control, functionalization or blending [3], allowing in turn different processing methods and different applications. Nevertheless, the most important aspects of their biomedical potential are the inherent biocompatibility and the ability to be degraded in a physiological environment into monomers which are naturally occurring metabolites [4]. When compared to the commercially available polyesters that have entered clinical practice (e.g., polylactic acid, PLA, or polylactic-*co*-glycolic acid, PLGA), PHAs show much more favorable degradation profiles, and their degradation products are not so acidic and do not cause local acidification of the environment or chronic inflammation [5,6,7,8,9]. In addition, they can be produced in a sustainable way using a variety of waste streams as the carbon source [10]. One of the main drawbacks in the biomedical application of PLA is its production, which although sustainable and cost-effective, still competes with food sources. Another widely investigated polyester for biomedical applications is polycaprolactone, PCL. PCL is a biodegradable, thermoplastic, synthetic polymer, but in contrast to PHAs which are bio-based, PCL is synthesized from crude oil and belongs to petroleum-based polymers. To overcome the problems associated with high costs and the usage of non-renewable feedstock, PCL is often blended or copolymerized with other bio-based polymers [11]. In addition, synthetic polyesters lack structural diversity and, hence, the possibility to modulate the properties to specific applications. Consequently, the wide range of PHAs’ biomedical applications includes drug delivery systems [3,6,12], medical implants and tissue engineering scaffolds [12,13,14,15]. The main limitations in the wider biomedical application of PHAs are high production costs and the lack of activity. In some instances, it is desirable that a biomaterial actively interacts with the organism inducing, in turn, certain effects (e.g., antimicrobial or cytotoxic activity, cell proliferation or inducing and maintaining a certain cellular phenotype). To achieve this, PHAs can be processed in a way to obtain biomimetic materials (electrospinning) or be combined with constituents with confirmed biological action, such as nanoparticles [16], antibiotics [17,18] or anticancer compounds [19,20].

**Figure 1:**
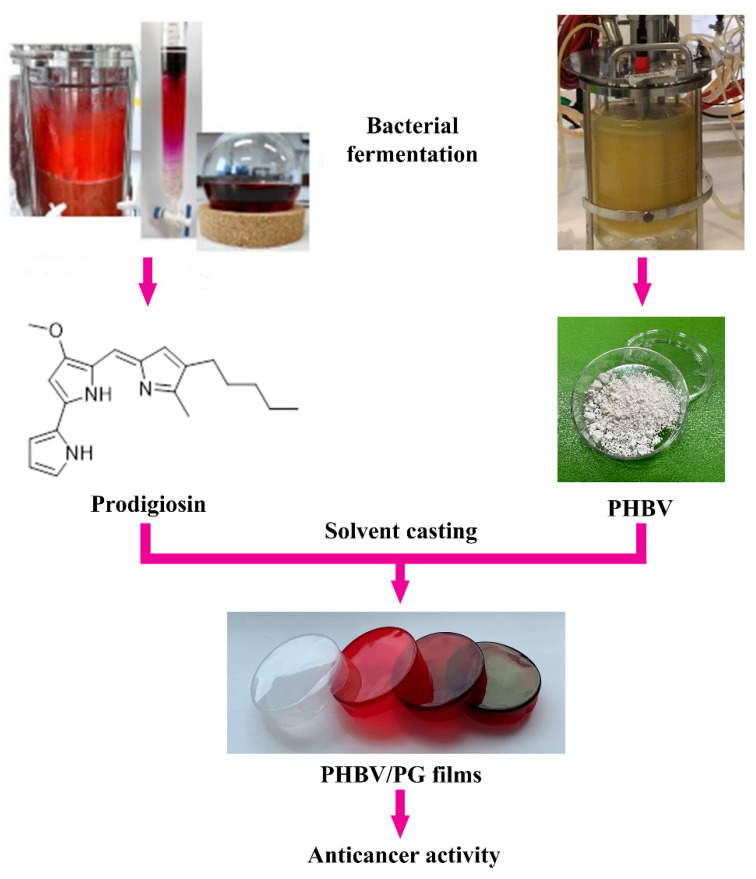
Study design and potential biomedical application of the PHBV films with incorporated prodigiosin (PG).

Prodigiosin (PG, C_20_H_25_N_3_O, Figure 1) is a bacterial biopigment produced by the secondary metabolism of both Gram-positive and Gram-negative bacteria, including several *Serratia* spp. [21,22]. For years, this molecule with a tripyrrolic core structure has been the focus of many studies that assessed its biological activity [23], prevalently in the field of anticancer drug research, but its immunosuppressive, antioxidative, UV protective, antimicrobial, antiparasitic and other properties were also explored [24,25]. PG entered pre-clinical trials for the first time in 2007 for the treatment of pancreatic cancer [24,26,27] but has not reached the market yet. However, the anticancer mechanisms of action and the specific molecular targets of PG are quite diverse [28,29]. Similar to PHBV, PG can be produced sustainably using bacterial fermentation [30,31]. It is also pH-responsive [32]. Thanks to these remarkable biological properties, PG can be combined with PHAs to develop new PHA-based formulations with enhanced functionality. This approach has been previously employed for the fabrication of PG-containing nanocomposites [31,33].

Here, the synthesis and characterization of biocompatible and bioactive biomaterials, in which both structural and functional constituents are of bacterial origin, are reported. These novel materials are based on bacterial polyester PHBV and bacterial pigment PG and are produced using a simple solvent casting method (Figure 1). The effect of PG incorporation on the physical properties (morphology, biopolymer crystallinity and thermal stability) and functionality (anticancer activity) of the obtained biomaterials was also investigated. Notably, a coloring effect of the biomaterials was also observed, which also provides a basis to further examine this and other bacterial natural products as suitable alternatives to chemical colorants.

## 2. Results

### 2.1. Film Morphology and Thickness

PHBV films with incorporated bacterial-pigmented natural product PG were successfully produced using the solvent casting method. The sample abbreviations and corresponding composition are given in Table 1.

The presence of PG within the biopolymer has affected the thickness of the obtained samples (Table 1). All pigment-containing samples were thinner in comparison to the PHBV control, and this effect was proportional to the PG content. Only the sample with the highest PG loading was somewhat thicker in comparison to the other formulations with lower pigment content, but this value was still lower than the control.

**Figure 2:**
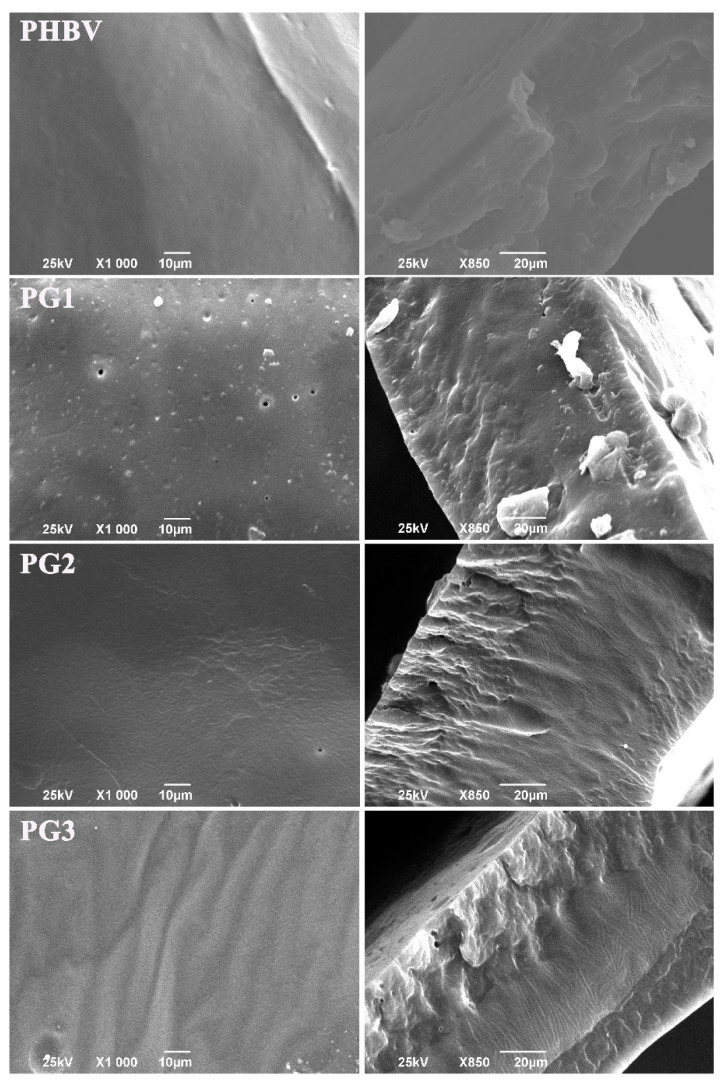
SEM micrographs of the surface and corresponding cross-sections of the neat biopolymer (PHBV) and the bacterial pigment containing (PG1, PG2 and PG3) films.

Morphology evaluation of the surface and the internal structure using SEM (scanning electron microscopy) has shown that the samples with PG exhibited heterogeneous cross-sections obtained by cryo-fracture (Figure 2). The higher the PG content, the more structured and rougher the surface of the fracture was. In contrast to the aforementioned, the neat biopolymer sample had an irregular, but not-so-structured, internal structure (Figure 2). The surface of the neat biopolymer was smooth, while the samples with PG appeared porous with distinctive topographical features.

### 2.2. Fourier Transform Infrared Spectroscopy (FTIR)

**Figure 3:**
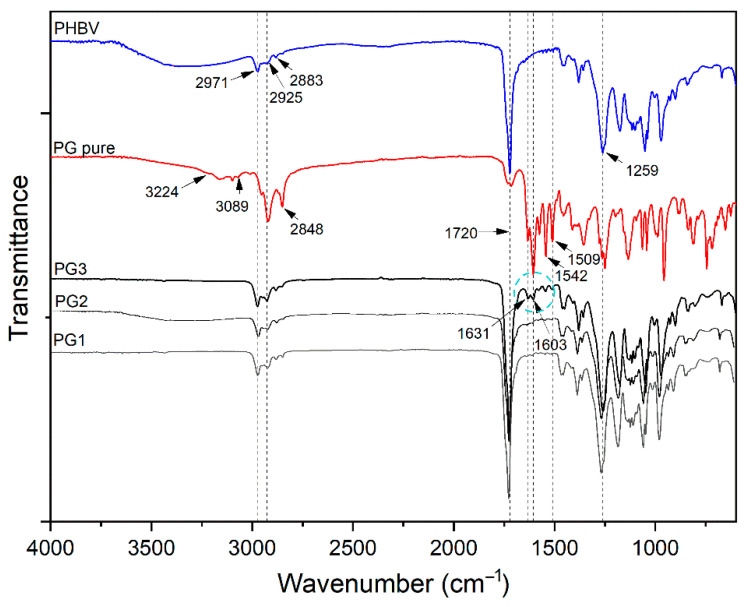
FTIR analysis of PHBV, pure biopigment prodigiosin (PG pure) and PG1–3 films.

FTIR-ATR spectroscopy was used to confirm the incorporation of PG into the PHBV polymer and the successful preparation of PHBV-PG films. Therefore, spectra of synthesized PHBV, pure PG and appropriate films were collected, and the results are shown in Figure 2. The most prominent bands in the PHBV spectrum appear at 2971 cm^−1^, 2925 cm^−1^, 2883 cm^−1^ and 2850 cm^−1^, corresponding to the C–H stretching modes of methine, methylene and methyl groups [34]; a strong absorption band at 1720 cm^−1^, attributed to the stretching vibration of the carbonyl group [35]; bands at 1381 cm^−1^ and around 1450 cm^−1^ from the symmetric wagging and bending mode of methyl groups, respectively [34]; and a strong band at 1259 cm^−1^ coming from the asymmetric stretching of saturated ester (C–O–C) bond. The most important bands in the spectrum of pure PG are broad peaks in the 3224–3089 cm^−1^ region and a band at 1603 cm^−1^ that can be attributed to secondary N–H bonds; peaks at 2953 cm^−1^, 2920 cm^−1^ and 2848 cm^−1^ from C–H stretching; a strong band at 1631 cm^−1^ from stretching of the –C=N– bond; and at 1542 cm^−1^ from stretching of the C=C double bonds [36]. Finally, the spectrum of the PG3 sample includes all mentioned bands from the PHBV polymer and pure PG spectra, as labeled in Figure 3.

### 2.3. Differential Scanning Calorimetry and Thermogravimetric Analysis (DSC/TGA)

**Figure 4:**
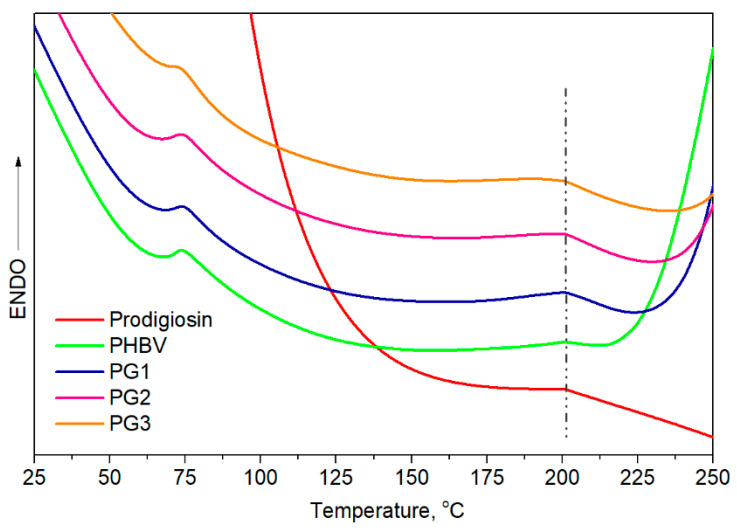
DSC curves of prodigiosin, pure PHBV and biopolymer films with incorporated PG (PG1–PG3).

Differential scanning calorimetry provided information about the thermal properties, melting temperature, *T*_m_, and melting enthalpy, Δ*H*_m_. From the obtained thermograms (Figure 4), two endothermic melting peaks were observed, and all samples had very similar *T*_m_ values, at around 75 °C and at 200.0 °C. The melting temperature of pure PG was calculated to be 201.0 °C, very close to *T*_m2_ of PHBV, making it difficult to predict whether PG was incorporated in an amorphous form or a crystalline form due to the overlapping of endothermic peaks. The calculated melting enthalpies were very low. The obtained Δ*H*_m_ of pure PHBV was quite low, at 13.3 J/g, in comparison to higher Δ*H*_m_ values of the samples with incorporated PG, from 22.9 J/g for PG1 to 26.5 J/g for PG3 (Δ*H*_m_ expressed as (Δ*H*_m1_ + Δ*H*_m2_), Table 2), indicating the contribution of PG as a nucleation-promotion agent in PHBV crystallization.

**Figure 5:**
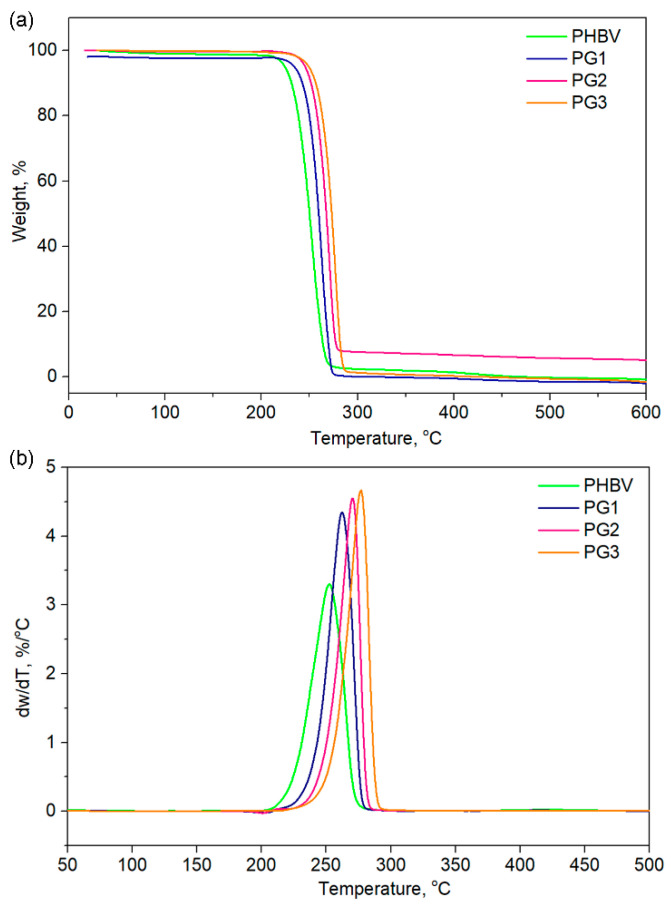
(**a**) TGA and (**b**) DTGA of the pure PHBV biopolymer and film samples with incorporated prodigiosin (PG1–3).

Thermogravimetric analysis (TGA) gave an insight into the thermal stability of the investigated samples and also revealed the influence of PG incorporation on the thermal properties of PHBV (Table 2, Figure 5). The TGA curves of the PHBV and PG samples with different amounts of PG are presented in Figure 5 for different characteristic degradation temperatures (*T*_5%_, *T*_10%_, *T*_50%_, *T*_90%_). Remarkable differences in the characteristic degradation temperatures of the PG films in comparison to starting PHBV were noticed even for a small weight loss of 5% (*T*_5%_).

The *T*_5%_ of PHBV was 224.0 °C, while the incorporation of PG resulted in increased thermal stability, hence the samples had higher values (from 239.0 °C for PG1 to 250.0 °C for the PG3 film, Table 2). This trend was observed for all the selected temperatures. The maximum degradation of the investigated samples was detected in the temperature range of 253.3 °C (PHBV) to 277.1 °C (PG3), as listed in Table 2. A total of 90% of the polymer sample mass was degraded at 263.8 °C for PHBV, at 269.9 °C for PG1, at 277.1 °C for PG2 and at 273.2 °C for PG3, clearly indicating that PG incorporation enhanced thermal stability of the obtained film samples (Table 2). Derivative thermogravimetric analysis additionally confirmed that PG had a significantly great influence on the maximum degradation temperatures, as well as on the degradation rate, while the degradation mechanism remained unchanged and the degradation proceeded in one step (Figure 5), as known for the PHBV-based polymers [37].

### 2.4. X-ray Diffraction (XRD)

**Figure 6:**
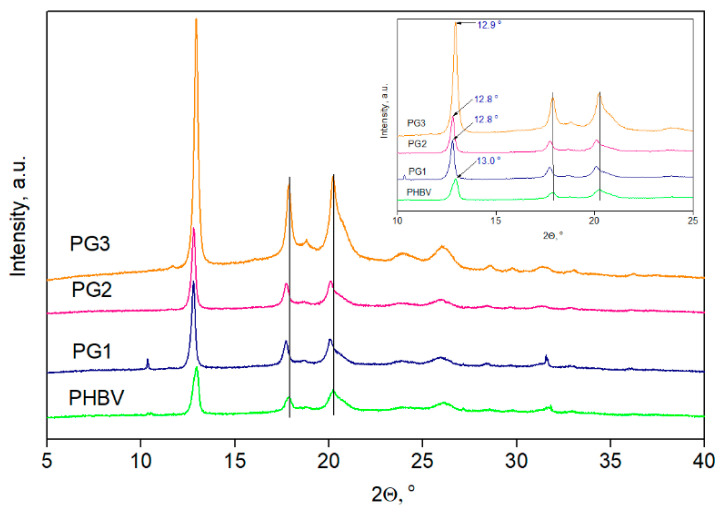
XRD diffractograms of PHVB and biopolymer films with incorporated prodigiosin (PG1–PG3).

The XRD diffractograms of the PHBV film and PG1-3 films are presented in Figure 6, while the degree of crystallinity, *X*_c_, is listed in Table 3.

In all recorded diffractograms, five PHBV characteristic peaks were detected at *2θ* values of 12.9°, 17.8°, 20.2°, 26.1° and 31.7° corresponding to the planes of (020), (110), (021), (121) and (200), respectively [38]. Additional peaks, visible in the PG3 sample diffractogram at 2*θ* values of 18.8° and 23.9°, could be attributed to the PG that was incorporated in the highest percentage in this film sample. After the PG incorporation into PHBV, the peak intensity increased, and this effect was accompanied by a shift in the characteristic peaks to lower 2*θ* values, indicating changes in the dimensions of the crystal lattice. Due to difficulties with the sample preparation, the diffractogram of pure PG is missing, but the XRD was applied to observe the changes in crystallization properties, as well as to estimate the influence of PG on the degree of crystallinity of the PHBV biopolymer matrix. The calculated value of the PHBV copolymer was 53%, while the PG1, PG3 and PG3 films had *X_c_* values of 64.0%, 63.9% and 69.2%, respectively (Table 3). Higher values of the degree of crystallinity for the examined films were due to the incorporation of PG, which acts as a nucleation agent and promotes the crystallization of PHBV.

### 2.5. Anticancer Potential

**Figure 7:**
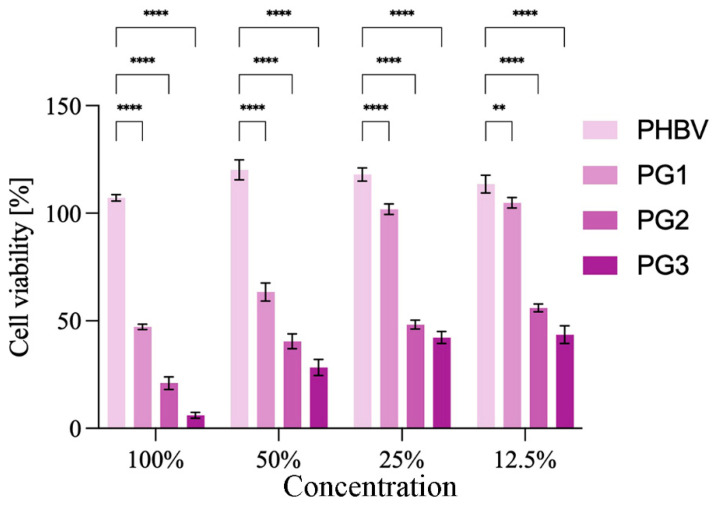
Anticancer studies: cytotoxicity of the obtained films towards HCT116 (colon cancer) cells are presented as a function of biomaterial composition (PG content) and biomaterial extract dilution. Significant differences compared to the control, neat biopolymer sample, for the same dilution are indicated by an asterisk (** *p* ≤ 0.01, **** *p* ≤ 0.0001).

As a compound with anticancer properties, PG represents an attractive candidate for incorporation into therapeutic biomaterials and their development for cancer treatment [39]. The anticancer potential of the obtained PHBV/PG films was evaluated using HCT116 (colon cancer) cells. The results are presented in Figure 7. Treatment with PHBV did not have any effect on the cancer cells. In contrast to this, the addition of PG to the biopolymer matrix improved the antiproliferative effect of the obtained films (Figure 7). All samples containing PG exhibited statistically significantly higher anticancer effects against cancer cells in comparison to the PHBV control. In addition, this effect was concentration dependent. A better anticancer potential was observed when the PG content was higher and when the material extract was more concentrated. Both the PG2 and PG3 samples exhibited excellent anticancer activity at all treatment dilutions, while PG1 (the film with the lowest PG content) was more efficient at higher concentrations (100% and 50%).

## 3. Discussion

The development of sustainable biomaterials from natural resources for application in the fields of biomedicine is of high importance, both for the circular economy and for the bioeconomy. The primary role of the drug delivery system is to provide controlled drug release in terms of time and location, and at the same time, to protect the drug from premature metabolic degradation, increase its bioavailability, prevent fluctuation in drug concentration and local or systemic toxicity and to ensure patient compliance [40]. Due to their proven biocompatibility and degradable nature, PHAs are ideal candidates for drug delivery systems [41]. So far, different PHAs, either in the form of drug-eluting films [42], microspheres [16,43,44] or nanoparticles [20,45,46], have been developed for controlled or targeted drug delivery [19]. Moreover, PHBV appeared as an innovative candidate for core-sheath nanofiber production suitable for bone tissue engineering [47,48]. Two biomolecules with remarkable biological properties, PHBV and PG, both obtained using bacterial fermentation, were combined in this study to obtain biocompatible, biodegradable and sustainable therapeutic biomaterials. For this stage of research, when PG was incorporated for the first time into the PHBV polymer matrix, the polymer film formulations obtained using solvent casting were chosen because it is a simple and cost-effective production method. This was a feasibility study in which we wanted to test the hypothesis of whether the incorporation of the active compound PG into PHBV is a good strategy to produce bioactive, biodegradable and sustainable biomaterials for potential biomedical application. Indeed, the fabrication procedure resulted in film samples with improved physical properties and functionality in comparison to PHBV alone.

The incorporation of PG into the PHBV matrix has affected both the thickness and morphology of the PHBV/PG samples. Besides being more compact (Table 1), the samples with PG exhibited more heterogeneously fractured cross-sections in comparison to the neat biopolymer, indicating a more ordered internal structure and higher degree of biopolymer crystallinity (which was further confirmed using XRD analysis). Similar findings were reported for films made from mcl-PHA, grafted with methyl acrylate (PHA-*g*-MC), where it was observed that PHA-*g*-MC films displayed more structured surface and cross-sections because grafting affects the amorphous nature of PHA [49]. Similarly, the study of poly(3-hydroxybutyrate) and poly(3-hydroxyoctanoate) blends has shown a correlation between a higher degree of crystallinity and more organized internal structure [50]. The increase in crystallinity of biopolymer films when mixed with another constituent has also been reported for chitosan films, where the addition of polyphenols has led to more regular packaging of the polymer chains [51]. In this work, the apparent change in biopolymer crystallization behavior in the presence of PG can also explain the difference in the sample surfaces. It seems that the smooth appearance of the neat PHBV can be explained by the slow crystallization rate which prevented pore formation, while the introduction of PG into the biopolymer matrix influenced biopolymer chains organization and crystallization, affecting, in turn, its surface properties. The subtle differences in the surface morphology observed among the different films containing increasing amounts of PG (PG1-PG3) have probably been caused by the different solvent evaporation rates from the film solutions with increasing PG content. Namely, a higher amount of PG resulted in a higher solution viscosity and furthermore changed the solvent evaporation rate, which finally influenced the films’ surface morphologies [52,53].

All absorption peaks inherent to the polymer repeating units, 3HV (3-hydroxyvalerate) and HB (3-hydroxybutirate), were detected in the FTIR-ATR spectrum of PHBV. The spectrum of pure PG confirmed the characteristic tripyrrole ring structure [54]. In addition to all the peaks from the PHBV polymer, the appearance of peaks at 1631, 1603, 1542 and 1509 cm^−1^ and the increasing intensity of the peak at 2925 cm^−1^ in the PHBV/PG spectrum undoubtedly confirm the incorporation of PG. Furthermore, the lack of peak shifts indicated that PG was not chemically bound, but only physically trapped inside the polymer backbone. The disappearance of bands in the 3224–3089 cm^−1^ region and decreasing and broadening of the peak at 1603 cm^−1^ implied the formation of hydrogen bonds between PG and the PHBV biopolymer. Although other characterization techniques used in the present work undoubtedly confirmed the incorporation of PG into all PHBV films, due to the lower percentage of PG used to produce PG1 and PG2 films (1.67 and 3.33 wt%, respectively), changes in their FTIR-ATR spectra were not noticeable.

Knowing that the polymer composition of PHBV preferentially affects its thermal properties, the first endothermic melting peak, assigned as *T*_m1_ at 75 °C, refers to the HV fraction, while the second endothermic peak detected at a higher temperature, *T*_m2_ at 200 °C, corresponds to the HB fraction. Despite the similar melting temperatures observed for all samples, the melting enthalpies of the PG-incorporated PHBV films were higher in comparison to pure PHBV and increased with the increase in PG amount. According to the obtained values, PG apparently acts as a nucleation agent that promoted the crystallization of PHBV.

The TG/DTG analysis indicated that PG was of great significance in terms of thermal stability and the degradation behavior of the PHBV polymer. Characteristic degradation temperatures gradually increased with the increase of dispersed PG concentration in the biopolymer matrix. The highest amount of incorporated PG resulted in the highest thermal stability of the PG3 film with *T*_max_ 277.1 °C. Although the degradation behavior of all PG-incorporated films was highly dependent on the amount of the bacterial pigment, the thermal decomposition occurred through a one-step degradation mechanism, most likely due to the random chain scission by *β*-elimination [55]. Furthermore, the incorporation of PG into the PHBV matrix significantly improved its thermal stability by 20 ^°^C, in comparison to the neat PHBV, but the degradation rate was also increased, with the highest degradation rate noticed for the PG3 sample, while the degradation rate value was notably lower for the neat PHBV sample.

XRD analysis revealed the crystalline pattern of the PHBV used for PG incorporation and also detected changes in the crystallinity caused by the addition of PG. The detected characteristic peaks corresponding to crystal planes (020), (110), (021), (121) and (200) and the unit cells of 3HB and 3HV in PHBV refer to orthorhombic PHBV crystal lattice, which is in agreement with the proposed pattern [56,57]. Shifting of the diffraction peaks indicated changes in the dimensions of the crystal lattice after PG incorporation into the PHBV polymer matrix. In order to quantify those changes, crystal interplanar distances (*d*-spacing) (three different planes of lattice were taken into account: planes (020), (110), and (021)) were calculated using Bragg’s equation [58,59]:

(1) λ=2d×sinθ

where *d* is the *d*-spacing, *λ* corresponds to the X-ray wavelength (1.5418 Å), and *θ* is the scattering angle. In addition, Scherrer’s equation [60] was used to calculate the crystallite size, *D* (nm):

(2) D=Kλβcosθ

where *K* is a dimensionless shape factor and usually taken to be 0.9, *λ* is the X-ray wavelength (0.154178 nm), *β* is the broadening of half the maximum intensity (FWHM) expressed in radians, and *θ* is the diffraction angle. According to all data listed in Table 3, shifting in the characteristic peaks of (020), (110) and (021) planes to lower values in comparison to PHBV indicated the increase in dimensions of crystal lattice which was further confirmed using the calculated *d* values [61]. Moreover, the *d*-spacing values (distance between atomic layers in the crystal) of the PHBV samples with incorporated PG increased for all calculated peaks, with the most prominent change confirmed for the peak at 2*θ*~13° where the *d* values increased from 6.67 nm to 6.91 nm. Referring to the crystal size, *D*, an increase in *D* values was detected for all PG-containing samples, with the highest increase calculated for the PG1 sample (from 23.5 nm to 44.47 nm, considering (020) plane), while these changes were less prominent in the case of the PG3 film (from 23.54 nm to 26.70 nm), which has the highest PG content. A not-so-prominent increase in *D* values of the PG3 films might be explained by the highest crystallinity in the series (69.2%) that resulted in a smaller crystallite size. The pure PHBV film had *X_c_* values of 53.0%, while the crystallinity of samples with the biopigment increased with the incorporation of PG in a concentration-dependent manner. Actually, PG acts as a nucleating agent promoting better organization and packaging of PHBV biopolymer chains forming higher-order crystalline structures, which is in agreement with some previous studies [62].

Anticancer studies have shown that samples with PG exhibit strong anticancer activity against HCT116 (colon cancer) cells, and this effect was concentration dependent. Compounds used in cancer therapies are usually highly toxic and hydrophobic, so PHAs are suitable carriers for this type of drug delivery system. An example of the PHBV-based system is nanoparticles loaded with elipticin, where encapsulation of the chemotherapeutic has led to the improved bioavailability and effectiveness of the incorporated drug [20]. Similar findings were reported for core-shell nanoparticles loaded with cisplatin [19]. Multiple studies have shown that PG actively inhibits the proliferation, migration and invasion of multiple cancer cell lines [63,64]. Nevertheless, its application is limited because its high hydrophobicity decreases its bioavailability [65]. To address this, the best approach to use PG as a therapeutic agent is to incorporate it into a carrier matrix. To date, PG was successfully encapsulated into chitosan [66] and poly lactic-*co*-glycolic acid (PLGA) [67] microspheres using emulsion techniques, which significantly enhanced its bioavailability and decreased breast cancer cell viability upon treatment. Hybrid composite PLGA/gelatin/pluronic F 127 electrospun fibers with PG exhibited not only prolonged cytotoxic effect against human breast cancer cells but also had smaller diameter when compared to the polymer fibers alone, indicating the influence of PG on polymer crystallization and fiber solidification [68], which was also observed in this work.

## 4. Materials and Methods

### 4.1. Materials

The precursors used for biomaterial production were chloroform (CHCl_3,_ Lach:ner, Neratovice, Czech Republic), polyhydroxyalkanoate and bacterial pigment PG. Both polyhydroxyalkanoate and PG were obtained from bacterial fermentation. Polyhydroxyalkanoate, in the form of a PHBV copolymer with high valerate content and a molecular weight of 5.4 × 10^5^ kDa, was produced as described before [69]. The PG-producing strain *Serratia marcescens* ATCC 27,117 was purchased from American Type Culture Collection (ATCC, Manassas, VA, USA). PG was produced using a bioreactor (Bio4, EDF-5.4_1, Biotehniskais centras AS, Riga, Latvia) as described previously [70] and extracted from bacterial cells to obtain the crude biopigment extract, which was further purified using gravitation column chromatography on silica gel.

### 4.2. Preparation of the Pigment-Containing Films

Biomaterials with incorporated bacterial pigments were produced in the form of films using the solvent-casting method. PHBV and PG were added to CHCl_3_ and stirred at room temperature in the dark for 30 min to ensure complete dissolution. The obtained film solutions were cast into glass Petri dishes (3 cm in diameter) and left to dry for 14 days at room temperature, protected from the sunlight.

Pure biopolymer film was prepared as described above, except that no pigment was added to the film solution. It served as a control. Samples were denoted as PHBV for the neat biopolymer film and PG1, PG2 and PG3 for the samples with increasing PG content. Sample abbreviations and corresponding compositions are presented in Table 1.

### 4.3. Material Characterization

#### 4.3.1. Film Thickness

Film thickness was measured at five random points of each sample formulation using a digital micrometer (0.001 mm accuracy) (S00014, Mitutoyo Corporation, Kawasaki, Japan).

#### 4.3.2. Scanning Electron Microscopy (SEM)

SEM imaging was performed using JEOL JSM-6390LV SEM (JEOL USA Inc., Peabody, MA, USA) operated at 25 keV. To examine the internal structure, the samples were cryo-fractured using liquid nitrogen. Prior to analysis, the samples were coated with a conducting layer of gold.

#### 4.3.3. Fourier Transform Infrared Spectroscopy (FTIR)

FTIR was conducted using a FTIR IRAffinity-1 spectrometer (SHIMADZU, Kyoto, Japan) at room temperature using the attenuated total reflectance (ATR) technique. The spectral range was 4000–600 cm^−1^ and the resolution was 4 cm^−1^.

#### 4.3.4. Differential Scanning Calorimetry and Thermogravimetric Analysis (DSC/TGA)

The DSC measurements coupled with TGA were performed using a TA Instruments SDT Q600 instrument in an atmosphere of nitrogen with a heating rate of 10 °C/min using standard aluminum crucibles. The applied temperature range for all samples was 25 to 600 °C, while the weight of the samples was about 5 mg.

The melting enthalpy obtained with DSC thermograms was determined from the area under the endotherms using TRIOS Software 5.2 TA Universal Data Analysis.

#### 4.3.5. X-ray Diffraction (XRD)

The crystal structure of PHBV/PG blended films was performed using an X-ray diffractometer (XRD) Rigaku Ultima IV, Japan, with CuKα1 radiation (*λ* = 0.154178 nm). The X-ray diffraction data were collected in the 2*θ* range from 10° up to 60° with the step of 0.02° and scanning rate of 5°/min. The degree of crystallinity of the investigated films was calculated using PeakFit 4.12 software.

### 4.4. Evaluation of Anticancer Potential

Investigation of the anticancer potential of the biopolymer films with PG was conducted using HCT116 (colon cancer) cells obtained from American Type Culture Collection (ATCC, Manassas, VA, USA), according to the previously described protocol [71]. In brief, 100 mg of the film sample was aseptically ground and incubated in 10 mL of RPMI-1640 medium for 72 h at 37 °C under constant shaking (180 rpm). Monolayer cell cultures were then treated with 50%, 25% and 12.5% (*v*/*v*) of a filtered film extract and incubated for 48 h. Cell proliferation was determined using standard MTT assay.

### 4.5. Statistical Analysis

GraphPad Prism 9.4.1 software (GraphPad Prism 9.4.1, La Jolla, CA, USA) was used to perform a two-way analysis of variance (ANOVA), followed by Tukey’s multiple comparisons test. The results are presented as mean ± standard deviations (SD). The difference was considered to be statistically significant at *p* ≤ 0.05.

## 5. Conclusions

In conclusion, bacterial pigment PG was successfully incorporated into the biocompatible and biodegradable bacterial polyester, PHBV. The obtained samples were in the form of a film and produced using a simple solvent casting method. The presence of PG, apart from giving lively color, has improved the physical properties and functionality of the obtained biomaterials. PG has acted as a nucleating agent, affecting, in turn, the degree of crystallinity, thermal stability, and morphology of the films. All samples with PG exhibited a more organized internal structure and had a higher degree of crystallinity and higher melting and degradation temperatures. This effect was proportional to the PG concentration. In addition, the films were also cytotoxic against colon cancer cells, indicating that the obtained PHBV/PG biomaterials can be potentially used in anticancer therapy.

## Figures and Tables

**Table 1:** Film sample abbreviations and corresponding compositions.

Sample	Biopolymer [mg]	Prodigiosin [mg]	CHCl_3_ [mL]	% wt ^1^	Thickness [µm]
PHBV	150	-	5	-	122.4 ± 4.0
PG1	150	2.5	5	1.67	117.6 ± 1.8
PG2	150	5	5	3.33	115.6 ± 3.5
PG3	150	10	5	6.67	118.8 ± 2.4

^1^ Pigment content relative to the biopolymer weight.

**Table 2:** DSC and TGA/DTGA (derivative thermogravimetric analysis) results of the PHBV, pure PG and film samples.

Sample	*T*_m1_, °C	*T*_m2_, °C	Δ*H*_m1_, J/g	Δ*H*_m2_,J/g	*T*_5%_, °C	*T*_10%_, °C	*T*_50%_, °C	*T*_90%_, °C	*T*_max_, °C
PHBV	74.8	200.4	10.81	2.46	224.0	231.2	250.0	263.8	253.3
PG	-	201.4	-	27.2	-	-	-	-	-
PG1	75.3	200.7	7.6	15.3	239.0	243.9	260.0	269.9	262.7
PG2	74.9	200.3	8.6	18.5	245.6	251.1	267.7	277.1	270.5
PG3	74.2	200.2	3.5	23.0	250.0	256.1	273.2	282.1	277.1

**Table 3:** XRD analysis results: degree of crystallinity, *X*_c_, full width at half-maximum (FWHM) of the peak, *d*-spacing and crystallite size, *D*.

Sample	*X*_c_, %	FWHM	*d*, nm	*D*, nm
(020)				
PHBV	53.0	0.34	6.67	23.54
PG1	64.0	0.18	6.91	44.47
PG2	63.9	0.25	6.91	32.03
PG3	69.2	0.30	6.83	26.70
(110)				
PHBV	/	0.42	4.94	19.16
PG1	/	0.24	5.00	33.53
PG2	/	0.34	5.00	23.68
PG3	/	0.34	4.95	23.41
(021)				
PHBV	/	0.79	4.39	10.21
PG1	/	0.54	4.42	14.96
PG2	/	0.51	4.23	15.86
PG3	/	0.93	4.40	8.70

## Data Availability

The data presented in this study are available on request from the corresponding author.
